# Reply to Mistry et al. The Two Substrate Reduction Therapies for Type 1 Gaucher Disease Are Not Equivalent. Comment on “Hughes et al. Switching between Enzyme Replacement Therapies and Substrate Reduction Therapies in Patients with Gaucher Disease: Data from the Gaucher Outcome Survey (GOS). *J. Clin. Med.* 2022, *11*, 5158”

**DOI:** 10.3390/jcm12124017

**Published:** 2023-06-13

**Authors:** Derralynn A. Hughes, Patrick Deegan, Pilar Giraldo, Özlem Göker-Alpan, Heather Lau, Elena Lukina, Shoshana Revel-Vilk, Maurizio Scarpa, Jaco Botha, Noga Gadir, Ari Zimran

**Affiliations:** 1 LSD Unit, Royal Free London NHS Foundation Trust, University College London, London NW3 2QG, UK; 2 Addenbrookes Hospital, Cambridge CB2 0QQ, UK; patrick.deegan@addenbrookes.nhs.uk; 3 CIBER de Enfermedades Raras, IIS Aragon, 50009 Zaragoza, Spain; giraldocastellano@gmail.com; 4 Translational Research Unit, IIS Aragon, 50009 Zaragoza, Spain; 5 Lysosomal Disorders Unit and Center for Clinical Trials, O&O Alpan LLC, Fairfax, VA 22030, USA; ogoker-alpan@ldrtc.org; 6 Department of Neurology, New York University School of Medicine, New York, NY 10016, USA; heather.lau4@gmail.com; 7 Department of Orphan Diseases, National Research Center for Hematology, 125167 Moscow, Russia; elenalukina02@gmail.com; 8 Gaucher Unit, Shaare Zedek Medical Center, Jerusalem 9103102, Israel; srevelvilk@gmail.com (S.R.-V.); azimran@gmail.com (A.Z.); 9 The Faculty of Medicine, Hebrew University, Jerusalem 9112102, Israel; 10 Centre for Rare Diseases, Academic Medical Centre Hospital of Udine, 33100 Udine, Italy; maurizio.scarpa@asufc.sanita.fvg.it; 11 Takeda Pharmaceuticals International AG, 8152 Zurich, Switzerland; jaco.botha@takeda.com (J.B.); noga.gadir-david@takeda.com (N.G.)

Thank you for allowing us the opportunity to respond to the commentary from Mistry and colleagues (Comment: The two substrate reduction therapies for type 1 Gaucher disease are not equivalent) [1] in relation to our published article (Switching between Enzyme Replacement Therapies and Substrate Reduction Therapies in Patients with Gaucher Disease: Data from the Gaucher Outcome Survey (GOS) [2]). We have carefully reviewed the comments in relation to our manuscript and respond to them in detail below. In the title of the comment, Mistry et al. note that the substrate reduction therapies (SRTs) mentioned are not equivalent. It is correctly noted that Miglustat and Eliglustat have different licensed indications, and indeed, we were careful to highlight this in both the introduction and discussion of our paper. It is also important to note that the earliest data presented within our paper predate the marketing authorization of Eliglustat.

The Gaucher Outcome Survey (GOS; NCT03291223) is a disease-specific registry for patients with a confirmed diagnosis of Gaucher disease, regardless of treatment status or type of treatment, and the data from GOS are therefore retrospective and represent what did happen, without implying what should happen. It was not our intention to provide a comparison of the safety and efficacy of Eliglustat and Miglustat, only to describe available data relating to patients switching between enzyme replacement therapies (ERTs) and SRTs.

The commentary provides important information regarding the chemistry and pharmacology of the two available substrate reduction therapies (Miglustat and Eliglustat), which were beyond the scope of our paper and provide useful general context. The commentators focus on one aspect of the relative gastrointestinal adverse event profiles of these molecules, that of diarrhea. We do not contend this issue in our paper. For many patients receiving Miglustat, they are advised to avoid starch/sugar intake at the same time as taking Miglustat in order to avoid this adverse event related to disaccharidase inhibition. The SmPC (summary of product characteristics) for Eliglustat lists gastrointestinal symptoms as ‘common’. In our experience, these tend to be reflux, indigestion, and constipation.

We are grateful to the commentary authors for explaining the cardiological adverse events associated with Eliglustat; we noted only one switch decision related to a cardiological adverse event, that of a myocardial infarction in a patient receiving Eliglustat.

Mistry et al. suggest that we appeared to present biased data on the adverse events (AEs) that led to treatment switching. As noted, the data are a presentation of the information recorded in a retrospective database. If the data were not recorded, we could not present it. We did report that AEs were the reason for switch in 18/78 Miglustat patients and 17/33 Eliglustat patients. Some of the Miglustat patients made their first switch prior to entry into GOS, and therefore the reasons for the decision may not be recorded. Miglustat was approved by EMA on 20 November 2002; GOS was started after the marketing authorization of velaglucerase alfa (VPRIV™- Takeda) in 2010.

We did, however, combine the data for patients switching from SRT to ERT in whom a specific AE was recorded. Specific AEs given as a reason for switching from SRT to ERT were provided for 29/35 patients and included gastrointestinal symptoms (n = 5), skin-related AEs (n = 5), dizziness (n = 3), drug intolerance (n = 3), fatigue (n = 2), change in taste (n = 2), and one case each of chills, cough, disorientation, general malaise, fracture, headache, poor balance, purpuric dermatosis, and moderate thrombocytopenia.

We concur with the suggestion of advice previously recorded for female patients receiving an SRT wishing to become pregnant. We believe that it was appropriate to combine the data presented, as the advice is common to both SRT products, noting that only 3/55 records cite this as a reason to switch. In this paper, we did not focus on aspects of pregnancy, which are published elsewhere. Similarly, we agree that the relative penetration of the agents to the cerebral spinal fluid is important background information.

Regarding discussions with patients, Mistry et al. focus on oral therapy, Eliglustat, for type 1 Gaucher disease, and we would add that discussions with patients with type 1 Gaucher disease should also include ERT.

In conclusion, we believe that our paper did specifically and accurately describe the reasons that were reported by the investigators of GOS to have led to a switch of modality. The data presented were a factual record from the GOS. The data capture period was complicated by the approval timing of multiple products but also the shortage of Cerezyme in 2009/10, which triggered a number of switches followed by several switchbacks. We believe that it is reasonable to combine some of the data for the two SRTs in this manner because of the differentiator for these products of the oral administration (vs. intravenous route for ERT) and, in some cases, low numbers. As is shown, we also separately reported data when it was appropriate to do so.

The discussion section helps the reader to contextualize the data in terms of the limitations of their observational, real-world characteristics.

## Supplementary Materials

The following supporting information can be downloaded at: https://www.mdpi.com/article/10.3390/jcm12124017/s1, Membership of GOS steering committee.
