# Supplementary material for: Reply to Mistry et al. The Two Substrate Reduction Therapies for Type 1 Gaucher Disease Are Not Equivalent. Comment on “Hughes et al. Switching between Enzyme Replacement Therapies and Substrate Reduction Therapies in Patients with Gaucher Disease: Data from the Gaucher Outcome Survey (GOS). J. Clin. Med. 2022, 11, 5158”
Source: J Clin Med. 2023 Jun 13;12(12):4017. doi: 10.3390/jcm12124017 (PMC10299381; doi:10.3390/jcm12124017)
Supplement: Supplementary file 1 [file jcm-12-04017-s001.zip › jcm-2299432-supplementary.pdf]

Membership of GOS steering committee

Tanya Collin-Histed – patient advocacy

Patrick Deegan

Deborah Elstein

Diego Fernández-Sasso

Pilar Giraldo

Derralynn Hughes

Elena Lukina

Shoshana Revel-Vilk

Maurizio Scarpa

Ida Schwartz – SC Chair

Stephan vom Dahl

Ari Zimran

Özlem Göker-Alpan
